# Biofloc technology application in aquaculture to support sustainable development goals

**DOI:** 10.1111/1751-7915.12836

**Published:** 2017-08-14

**Authors:** Peter Bossier, Julie Ekasari

**Affiliations:** ^1^ Aquaculture & Artemia Reference Center Ghent University Rozier 44 9000 Ghent Belgium; ^2^ Department of Aquaculture Faculty of Fisheries and Marine Science Bogor Agricultural University (IPB) Kampus IPB Darmaga Bogor Jawa Barat Indonesia

## Abstract

Biofloc technology (BFT) application offers benefits in improving aquaculture production that could contribute to the achievement of sustainable development goals. This technology could result in higher productivity with less impact to the environment. Furthermore, biofloc systems may be developed and performed in integration with other food production, thus promoting productive integrated systems, aiming at producing more food and feed from the same area of land with fewer input. The biofloc technology is still in its infant stage. A lot more research is needed to optimise the system (in relation to operational parameters) e.g. in relation to nutrient recycling, MAMP production, immunological effects. In addition research findings will need to be communicated to farmers as the implementation of biofloc technology will require upgrading their skills.

Aquaculture as a food‐producing sector offers ample opportunities to alleviate poverty, hunger and malnutrition, generates economic growth and ensures better use of natural resources (Food and Agriculture Organization, 2017). Aquaculture production is projected to rise from 40 million tonnes by 2008 to 82 million tonnes in 2050 (FAO, 2010). The necessity to increase aquaculture production has been triggered by the increasing demand per capita in parallel to the increase of global population. However, the development of a sustainable aquaculture industry is particularly challenged by the limited availability of natural resources as well as the impact of the industry on the environment (Costa‐Pierce *et al*., 2012; Verdegem, 2013). With these limitations in mind, the development of sustainable aquaculture industry should focus on the conceptualization of systems that despite their high productivity and profitability, utilize fewer resources including water, space, energy and eventually capital, and at the same time has lower impact on the environment (Asche *et al*., 2008; FAO, 2017). Along with SDG 14 targets, sustainable aquaculture development could contribute to multiple objectives including ending poverty (SDG 1), ending hunger, achieving food security and improved nutrition (SDG 2) and promoting sustained, inclusive and sustainable economic growth (SDG 8) (Food and Agriculture Organization, 2017).

One of the strategies to improve aquaculture production and sustainability should focus on enhancing feed nutrient utilization. This can be developed by two different approaches, i.e. (i) by increasing the feed quality and feeding strategy in a way that the nutrients can be efficiently delivered and finally utilized and (ii) by re‐utilizing the nutrient waste through modifications in the culture system. In an aquatic system, nutrients can be removed by various natural biogeochemical processes involving mostly microorganisms with various functions in nutrient cycles. The nutrient waste in an aquaculture system is mostly generated from unconsumed feed and the digestion and metabolic processes of feed. Nutrient waste in an aquaculture system may be re‐utilized directly by other organisms at lower trophic levels, which utilize feed particles as their food source, or indirectly by the conversion of the nutrients into microbial biomass that may eventually be consumed by the cultured animal itself or other animal as their food source.

Biofloc technology is mainly based on the principle of waste nutrients recycling, in particular nitrogen, into microbial biomass that can be used *in situ* by the cultured animals or be harvested and processed into feed ingredients (Avnimelech, 2009; Kuhn *et al*., 2010). Heterotrophic microbiota is stimulated to grow by steering the C/N ratio in the water through the modification of the carbohydrate content in the feed or by the addition of an external carbon source in the water (Avnimelech, 1999), so that the bacteria can assimilate the waste ammonium for new biomass production. Hence, ammonium/ammonia can be maintained at a low and non‐toxic concentration so that water replacement is no longer required.

Biofloc technology enhances the production and productivity by its contribution to the supply of good quality fish juveniles, the latter being one of the most important inputs in the production. In addition, it contributes to the improvement of the fish production. In relation to the former, biofloc technology could support the supply of good quality seeds by improving the reproductive performance of aquaculture animals and by enhancing the larvae immunity and robustness (Ekasari *et al*., 2015; Ekasari *et al*., 2016; Emerenciano *et al*., 2013). In relation to the latter, the application of biofloc technology in grow out systems of some aquaculture species could improve net productivity by 8–43%, relative to the non‐biofloc control (traditional with water exchange, clear water system or recirculating aquaculture system) (Ekasari, 2014).

## Biofloc systems provide a nutritious food source and can improve feed utilization efficiency

*In situ* utilization of microbial flocs generated in biofloc systems by some aquaculture organisms as well as the utilization of processed bioflocs as a feed ingredient has been well documented (Kuhn *et al*., 2009, 2010; Anand *et al*., 2014). Ju *et al*. (2008) demonstrated that the concentrations of free amino acids such as alanine, glutamate, arginine and glycine, which are known attractants in shrimp diet (Nunes *et al*., 2006), are present in bioflocs. Levels in bioflocs were found to be comparable to that of the shrimp commercial diet suggesting that bioflocs are likely to be recognized as food particles by some aquaculture organisms. Furthermore, biofloc technology application in larviculture (at least to some species which can handle particles in suspension) may provide easily accessible food source for the larvae outside the regular feeding moments, thus minimizing possible negative social interaction during feeding (Ekasari *et al*., 2015).

Studies have demonstrated a more efficient dietary nutrient assimilation in this system. Da Silva *et al*. (2013) reported that the application of biofloc technology on Pacific white shrimp super intensive culture considerably enhanced N and P utilization efficiency up to 70% and 66%, respectively, relative to conventional intensive culture systems with regular water exchange. Another report by Avnimelech (2007) noted that applying biofloc technology in tilapia intensive cultures increased nitrogen recovery from 23% to 43%. Essentially, biofloc studies with Pacific white shrimp (Xu and Pan, 2012), tilapia (Azim and Little, 2008) and green tiger shrimp (Megahed, 2010) clearly showed the possibility to reduce protein content in the feed. Moreover, Ray *et al*. (2010) pointed out that the use of plant‐based diet (96% protein obtained from plant‐based ingredients) is favourable in a biofloc system. The reduction of protein content of the feed and the use of plant‐based protein sources in the feed are considered to be more sustainable and eco‐friendly because of the reduced production of nitrogenous and phosphorous waste. It also reduces the dependency on overexploited marine resources.

Bioflocs may contribute to the supply of essential nutrients and digestive enzymes either through the stimulation of endogenous production or microbial secretion (Xu and Pan, 2012; Anand *et al*., 2014), and the enhancement of nutrient bioavailability that facilitates higher nutrient assimilation. As a protein source, bioflocs could be considered as a good protein source for shrimp and a useful protein source for tilapia and mussel (Ekasari *et al*., 2014,b). Bioflocs also contain various bioactive compounds including essential fatty acids, carotenoids, free amino acids and chlorophylls (Ju *et al*., 2008), trace minerals (Tacon *et al*., 2002) and vitamin C (Crab *et al*., 2012) which are known to have positive effects on aquaculture animals including the enhancement of antioxidant status, growth, reproduction and immune response.

Bioflocs also offers a lot of MAMPs (microbial associated molecular patterns), which may be recognized as immunostimulants, resulting in higher resistance to diseases (Ekasari *et al*., 2014,b). Interestingly, when biofloc technology was applied in tilapia broodstock culture system, it enhanced the immunological status contributing to the improvement of the larvae robustness against diseases and environmental stress test (Ekasari *et al*., 2015, 2015; Ekasari *et al*., 2016). In biofloc systems, aquaculture animals may also benefit from reduced pathogen pressure. Some studies demonstrated that the presence of potentially pathogenic bacteria might be reduced in biofloc systems (Crab *et al*., 2010b; Zhao *et al*., 2012). It has been suggested that the reduction of *V. harveyi* population in biofloc environment might be related to the disruption of *V. harveyi* cell‐to‐cell communication also known as an important factor in determining the pathogenicity of this particular bacterium (Crab *et al*., 2010b).

## Biofloc systems reduce water utilization and waste generation

Equally important as target species production enhancement, the application of biofloc technology may significantly reduce the quantity of water used, a main resource in aquaculture. To illustrate, an intensive zero exchange lined shrimp pond only required 1–2.26 m^3^ kg^−1^ shrimp, whereas a conventional system with regular water exchange may require water up to 80 m^3^ kg^−1^ (Hargreaves, 2006). In addition, Luo *et al*. (2014) noted that water consumption of biofloc‐based tilapia culture system was 40% lower than that of recirculating aquaculture system (RAS).

Most of the studies applying biofloc technology confirmed that the N and P waste in this system could be reduced, corroborating the role of this system on the improvement of aquaculture productivity and the reduction of environmental impact from aquaculture unit (among others, Pérez‐Fuentes *et al*., 2013; Luo *et al*., 2014). Although heterotrophic bacteria are the main nitrogen conversion agent, biofloc system also facilitate other nitrogen conversion mechanisms including nitrification (Ekasari, 2014), phototrophic N uptake (Emerenciano *et al*., 2013) and denitrification (Hu *et al*., 2014) (all dependent on the prevailing environmental conditions). The nutrient recycling by the microbial loop involves the uptake of inorganic phosphorus by heterotrophic bacteria (Kirchman, 1994), which is not only reducing the discharged P, but also enhancing the bioavailability of this nutrient for the cultivated animals. The level of P assimilation efficiency of fishmeal and plant‐based ingredients by fish has been perceived to be limited by the high level of indigestible bone‐P and phytate‐P; therefore, it is likely that this nutrient will be egested in the faeces rather than utilized by the cultivated animals. The consumption of the microbial biomass in the biofloc might therefore facilitate P assimilation, in particular the indigestible one, from the feed to the cultivated organisms thus reducing the nutrient waste (Luo *et al*., 2014, Da Silva *et al*., 2013).

## Biofloc‐based integrated aquaculture system for higher productivity, higher nutrient utilization and lower aquaculture pollution

A possible modification in biofloc‐based aquaculture to maximize nutrient utilization efficiency is by the applying nutrient recycle principle in an integrated aquaculture system. The faster conversion of nutrient by the microbes associated in bioflocs or periphyton may provide more digestible and nutritious additional food source for both main cultured organism and other species added into the system. In this way, utilization of the wasted nutrients is expected to be more efficient and less pollution is generated. The recent study by Liu *et al*. (2014) showed that the addition of maize to stimulate bioflocs grown in an integrated culture of shrimp, spotted scat and water spinach significantly increased shrimp total yield, reduced total food conversion ratio (FCR) and lowered total P and total N in the cultured water. Interestingly, combining biofloc system with integrated multi trophic culture system may also enhance nutrient utilization efficiency. Ekasari (2014) demonstrated that combining biofloc‐based shrimp culture system with tilapia, mussel and seaweed resulted in higher production, higher feed N and P recovery by the shrimp and the entire culture system, and simultaneously resulted in reduced waste nutrient and microbial biomass. Furthermore, the addition of seaweed or macrophytes (Brito *et al*., 2014; Liu *et al*., 2014; Pinho *et al*., 2017) in a biofloc‐based integrated aquaculture system may also bring about the possibility to capture the excess CO_2_, which may result in an increase in C utilization efficiency and a reduction in the emission of GHG. This additional benefit in nutrient utilization efficiency should stimulate further research on the possibility of incorporating biofloc system into an integrated multitrophic culture system to mitigate negative environmental impact of aquaculture nutrient wastes.

## Conclusion

Biofloc technology application offers benefits in improving aquaculture production that could contribute to the achievement of sustainable development goals. This technology could result in higher productivity with less impact to the environment. Furthermore, biofloc systems may be developed and performed in integration with other food production, thus promoting productive integrated systems, aiming at producing more food and feed from the same area of land with fewer input. The biofloc technology is still in its infant stage. A lot more research is needed to optimize the system (in relation to operational parameters) e.g. in relation to nutrient recycling, MAMP production and immunological effects. In addition, research findings will need to be communicated to farmers as the implementation of biofloc technology will require upgrading their skills.

## Conflicts of Interest

None declared.
